# Molecular Signatures Discriminating the Male and the Female Sexual Pathways in the Pearl Oyster *Pinctada margaritifera*

**DOI:** 10.1371/journal.pone.0122819

**Published:** 2015-03-27

**Authors:** Vaihiti Teaniniuraitemoana, Arnaud Huvet, Peva Levy, Nabila Gaertner-Mazouni, Yannick Gueguen, Gilles Le Moullac

**Affiliations:** 1 Ifremer, UMR 241 Ecosystèmes Insulaires Océaniens (EIO), Labex Corail, Centre du Pacifique, BP 7004, 98719 Taravao, Tahiti, French Polynesia; 2 Ifremer, UMR 6539 Laboratoire des sciences de l’Environnement Marin (LEMAR), ZI de la Pointe du Diable, CS 10070, F-29280 Plouzané, France; 3 Université de la Polynésie Française, UMR 241 Ecosystèmes Insulaires Océaniens (EIO), Labex Corail, BP 6570, 98702 Faa'a, Tahiti, French Polynesia; 4 Ifremer, UMR 5119 Ecologie des Systèmes Marins Côtiers (ECOSYM), Labex Corail, Université de Montpellier, Place Eugène Bataillon, CC 80, F-34095 Montpellier Cedex 5, France; The Ohio State University, UNITED STATES

## Abstract

The genomics of economically important marine bivalves is studied to provide better understanding of the molecular mechanisms underlying their different reproductive strategies. The recently available gonad transcriptome of the black-lip pearl oyster *Pinctada margaritifera* is a novel and powerful resource to study these mechanisms in marine mollusks displaying hermaphroditic features. In this study, RNAseq quantification data of the *P*. *margaritifera* gonad transcriptome were analyzed to identify candidate genes in histologically-characterized gonad samples to provide molecular signatures of the female and male sexual pathway in this pearl oyster. Based on the RNAseq data set, stringent expression analysis identified 1,937 contigs that were differentially expressed between the gonad histological categories. From the hierarchical clustering analysis, a new reproduction model is proposed, based on a dual histo-molecular analytical approach. Nine candidate genes were identified as markers of the sexual pathway: 7 for the female pathway and 2 for the male one. Their mRNA levels were assayed by real-time PCR on a new set of gonadic samples. A clustering method revealed four principal expression patterns based on the relative gene expression ratio. A multivariate regression tree realized on these new samples and validated on the previously analyzed RNAseq samples showed that the sexual pathway of *P*. *margaritifera* can be predicted by a 3-gene-pair expression ratio model of 4 different genes: *pmarg-43476*, *pmarg-foxl2*, *pmarg-54338* and *pmarg-fem1-like*. This 3-gene-pair expression ratio model strongly suggests only the implication of *pmarg-foxl2* and *pmarg-fem1-like* in the sex inversion of *P*. *margaritifera*. This work provides the first histo-molecular model of *P*. *margaritifera* reproduction and a gene expression signature of its sexual pathway discriminating the male and female pathways. These represent useful tools for understanding and studying sex inversion, sex differentiation and sex determinism in this species and other related species for aquaculture purposes such as genetic selection programs.

## Introduction

The black-lip pearl oyster *Pinctada margaritifera* (Linnaeus, 1758) var. *cumingi* (Jameson, 1901) is a benthic bivalve occurring in subtropical and tropical coral reefs. It is widely distributed in the Indo-Pacific region: throughout the Equatorial zone, down to Northern Australia and extending up to the south of Japan [1].

For French Polynesia, the black pearl industry, based on the cultured of *P*. *margaritifera*, is the second most important economic resource. Over the last three decades, research projects have been conducted to support sustainable development of pearl farming and to improve the quality of the pearls produced [2], using genetic methods. Genetic selection programs have been encouraged because it was demonstrated that the pearl quality is dependent on the genetics of the graft donor oyster [3]. The aim of the *P*. *margaritifera* selection program, is to breed families of graft donor oysters selected for their capacity to produce high quality pearls and/or particular colors and/or rapid growth [4–6].

The successful hatching of selected spat depends on the production of gametes and embryos from synchronous selected breeders raised in laboratory conditions. Reproduction of pearl oysters under controlled conditions mainly depends on the availability of females, which are unfortunately not abundant among farmed oysters due to the protandrous hermaphroditism of this species [7]. As with other pearl oysters, *P*. *margaritifera* shows a consecutive switch in sex: individuals may change gender (from male to female) from the end of their second year onwards [8]. However, Thielley (1993) [9] showed that changes from female to male could occur when conditions, either natural (temperature or food) or unnatural (handling or cleaning), were stressful. These observations would better correspond to alternate hermaphroditism. In all cases, simultaneous hermaphrodites and animals with undetermined status were uncommon. Knowledge of the underlying physiological mechanisms and factors controlling sex ratio [8,10,11] especially for the female pathway, are essential to establish a genetic improvement program.

Sex ratio is the combined result of sex determination and sex differentiation. In the animal kingdom, sex determination, the process by which the sex (gender, male or female) of an individual is established, can be genetic (genetic sex determination, GSD), environmental (environmental sex determination, ESD), or result from an interaction of both of these factors [12]. These genetic and/or environmental sex determining switches lead to sex differentiation, the process by which specific molecular cascades transform an undifferentiated gonad into a testis or an ovary [13]. Although these mechanisms are well known in vertebrates, data on sexual determinism and sex differentiation in marine mollusks are still poorly understood. In bivalve mollusks, sex determination systems appear complex and dynamic, from diocy to hermaphroditism [8]. In the pacific oyster *Crassostrea gigas*, an alternative protandrous hermaphrodite bivalve, genetic models suggest that sex determination would be controlled by a major gene, which may be under environmental influence [14,15]. To date, several genes homologous to sex-determining pathway genes in model species have been identified in *C*. *gigas* such as *Cg-DMl* and *Cg-SoxE* homologous to male sex-determining pathway genes, and *Cg-β-catenin*, *Cg-foxl2* and its natural antisense transcript *Cg-foxl2os* homologous to female sex-determining pathway genes. Their expression profiles in this species strongly suggest their implication in male and female gonad differentiation but no evidence was found of a role in sex determination. However their expression profiles may imply the existence of a sex determination time window at the end of the gametogenetic cycle [16–20]. In most bivalves, the main environmental factors affecting reproduction, and probably gender, are temperature, food availability [21,22] and, to a lesser degree, photoperiod [23]. In the Pacific oyster *Crassostrea gigas*, Enríquez-Díaz *et al*. (2009) [24] showed that while intensity was influenced by the quantity of available food, the timing and speed of gametogenesis were regulated by temperature.

During recent years, genomics of marine bivalves of major economic interest has been studying in order to provide better understanding of the molecular mechanisms underlying reproduction, including reproductive cycle, sex differentiation and sex determination [25–28]. Recently, Teaniniuraitemoana *et al*. (2014) [29] performed a gonad transcriptome analysis of *P*. *margaritifera*, using Illumina-based RNAseq, unraveling some molecular mechanisms involved in sex determination, sex differentiation and gametogenesis. In this study, the authors revealed the importance of *pmarg-dmrt*, *pmarg-fem1-like* and *pmarg-foxl2* as starting points for further functional studies on the sex-determining cascade of the pearl oyster.

In the present study, using the previously published RNAseq dataset [29], we applied a more stringent method of statistical selection on the differential expression levels in order to draw up a short list of candidate genes of the male and the female sexual pathway. These candidates were next assayed by real-time PCR in different sexes and stages of pearl oyster gonad. Through these analyses, we obtained new insights into the reproduction of *P*. *margaritifera* based on a dual histo-molecular analytical approach, allowing a model to be built based on expression ratio of three gene pairs, and predictions of sexual pathway to be made for individuals of this hermaphrodite species. This study offers molecular signatures of the female and male sexual pathways that will be useful for future research on reproduction in *P*. *margaritifera*, especially research on sex differentiation and sex determination.

## Materials and Methods

### Ethics statement

The authorization (No. 4226) of the pearl oyster translocation from Takaroa atoll (14°26’59.12”S, 144°58’19.91”W, Tuamotu Archipelago, French Polynesia) to the Vairao lagoon (Ifremer marine concession No. 8120/MLD: 17°48’26.0”S, 149°18’14.4”W, Tahiti, French Polynesia) has been issued by the Ministry of Marine Resources on September 27, 2012 after the favorable notice given by the town councils of Takaroa and Vairao, respectively. At the departure of Takaroa atoll, pearl oysters were packed in ice-box for shipment by air. Upon their arrival at Vairao, pearl oysters were immersed for 30 min in a hyper-salted water bath (120 g of salt L^-1^ water) following the recommendations with the authorization for transfer in order to eliminate anemones and sea squirts. Then, the pearl oysters are stored in the lagoon of Vairao for 3.5 months to recover physiologically of air transport and hyper-salted treatment. In any case, this study involves protected or endangered species.

### Animal and tissue sampling

Three-year-old adult *P*. *margaritifera* of an average size of 118 mm ± 8.67 from Takaroa atoll, acclimated in the Vairao lagoon for three and a half months, were brought to the Ifremer laboratory in Tahiti, French Polynesia, in April 2013. For each oyster, gonad tissues were sampled, making a transversal section of the gonadic area for histological examination. A piece of the rest of the gonad was conserved in RNAlater (Qiagen) (50 mg mL^-1^) and stored at -80°C until RNA extraction.

### Histology

Gonad tissues of 44 oysters were sampled for histological examination using methods described by Fournier *et al*. (2012) [30]. After dissection, each visceral masse was fixed in formalin solution 10% diluted in seawater for 48 h before being transferred in ethanol at 70% for 48 h for preservation. Then the visceral masses were cut according to their sagittal plane with a microtome blade and were dehydrated through a graded ethanol series, embedded in paraffin, sectioned at 3–4 μm on a rotary microtome, stained with Giemsa dye and, finally, mounted on microscope slides. Gonad differentiation stage and sex were determined under a light microscope and samples were classified according to the different categories of gonadic tissues previously described in Teaniniuraitemoana *et al*. (2014) [29]: male and female at “Early” stage (gonad in early gametogenesis; Male: n = 4; Female: n = 3), “Intermediate” stage (gonad developing; Male: n = 4; Female: n = 4), at “Mature” stage (oyster ready to spawn; Male: n = 4; Female: n = 2), “Regressed” stage (gonad has stopped generating gametes; Male: n = 9; Female: n = 8); and “Undetermined” (gonad contains no gametes at all; n = 6). No gonads in “Inversion” (where the gonad presents both male and female gametes) were observed in this sample.

### Real-time PCR

Candidate marker genes selected from the analysis of RNAseq dataset were assayed by real-time PCR on the 44 gonad samples. After removing RNAlater by pipetting and absorption, total RNA of each individual was extracted using TRIzol Reagent (Invitrogen) and treated with DNAse I using a DNA-*free* Kit (Ambion) following manufacturer’s instructions. RNA concentrations were measured on an ND-100 spectrophotometer (Nanodrop Technologies) at 260 nm, using the conversion factor 1 OD = 40 μg mL^-1^ RNA. For each sample, 0.5 μg of total RNA were reverse-transcribed using Transcriptor First Strand cDNA Kit (Roche) and amplified by real-time PCR on a Stratagene MX3000P. The amplification reaction contained 12.5 μL 2X SYBR green qPCR Master Mix (Stratagene), 10 μL cDNA template, and 2.5 μL of primers (4 μM) in a final volume of 25 μL. Each run included a positive cDNA control and a blank control (water) for each primer pair. Relative gene expression was calculated using two reference genes, *ef1a* and *gapdh1*, by the 2^-ΔCt^ method [31], as follows: Relative expression_(target gene, sample x)_ = 2^^-^(Ct_target gene, sample x_—Ct_reference gene, sample x_). The relative stability of the *ef1a* and *gapdh1* combination, considering the sex and reproductive stage of each gonad sample, was confirmed using NormFinder [32] (Stability value for best combination = 0.288). PCR efficiency (E) was estimated for each primer pair by determining the slopes of standard curves obtained from serial dilution analysis of a cDNA to ensure that E ranged from 90 to 110%. The primers used for amplification are listed in S1 Table.

### Statistical analysis

All the statistical analyses were performed using R v2.15.2, an environment and a language for statistical computing [33].

#### Differential expression (DE) analysis

RNAseq quantification data of *P*. *margaritifera* gonads were taken from the study of Teaniniuraitemoana *et al*. (2014) [29] (Bioproject PRJNA229186—Genbank accession number SRP033217) who analyzed the gonad transcriptome based on the Illumina sequencing of 36 cDNA libraries, each corresponding to gonad samples of different reproductive stages and sexes.

The differential expression level of contigs between different gonadic categories was tested using the DESeq package v1.11.3 [34] as described in Teaniniuraitemoana *et al*. (2014) [29]. In the present study, the differential expression of contigs was considered as statistically significant when the value of *FoldChange* was > 10 or < 0.1 i.e., when there was a tenfold change in expression under one of the conditions; and a *padj* < 0.001, *p-value* adjusted with a false-discovery rate (FDR) correction for multiple testing by the Benjamini-Hochberg method [35].

#### Relative expression analysis

Normality and homoscedasticity of gene relative expression data were checked using Shapiro-Wilk and Bartlett’s tests. One-way ANOVA was performed followed by Tukey’s multiple comparison tests to determine expression differences of candidate marker genes between the gonadic categories considered.

#### Hierarchical clustering analysis

Hierarchical clustering was applied firstly to RNAseq samples and secondly to real-time PCR samples in order to cluster them according to the similarity of expression pattern of the statistically significant differentially expressed contigs and expression pattern of the selected candidate marker genes, respectively.

#### Decision tree

We adapted and performed a multivariate regression tree (MRT), widely used in the domain of ecology for modeling species-environment relationships [36], to evaluate the hierarchical importance of the effect of relative gene expression on the oyster sexual pathway. Divisions in the MRT were determined by cross-validation. We performed these analysis with the mvpart package v1.6–1 [37] using the default parameters of the *rpart* function.

## Results

### Differential expression analysis reveals new insight into *P*. *margaritifera* reproduction

To provide an overall view of the transcriptional changes in the gonad of *P*. *margaritifera*, a stringent differential expression analysis was performed between the ten different gonadic categories on the RNAseq dataset already available [29]. In accordance with the value of *FoldChange* > 10 or < 0.1 and *padj* < 0.001, the DESeq method identified 1,937 contigs differentially expressed between gonadic categories.

**Fig 1:**
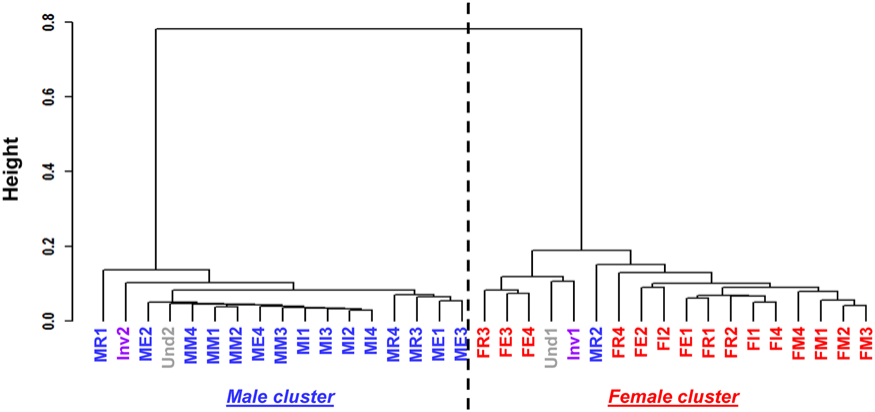
Hierarchical clustering using Spearman’s correlation on RNAseq gonad samples. Samples are divided into two clusters based on their contig expression pattern, discriminating male (blue) and female gonads (red). (M/F)E: male/female at early stage; (M/F)I: male/female at intermediate stage; (M/F)M: male/female at mature stage; (M/F)R: male/female at regression stage; purple or Inv: gonads in sexual inversion; grey or Und: gonad sex is undetermined.

A hierarchical clustering method was applied on the RNAseq samples to cluster them according to the similarity of expression pattern among the 1,937 differentially expressed contigs (Fig. 1). Analysis of the dendrogram generated identified two clusters: one male, gathering the samples of male sex, and one female, gathering the samples of female sex. Moreover, the hierarchical clustering put one of the two gonad samples of the “undetermined” category in each cluster and did the same with the two gonad samples in sexual inversion. The one male gonad sample in regression was classified with female gonads. Thus, Fig. 1 illustrates how the samples “Und1”, “Inv1” and “MR2” showed a female gene expression pattern whereas the samples “Und2” and “Inv2” showed a male gene expression pattern.

**Fig 2:**
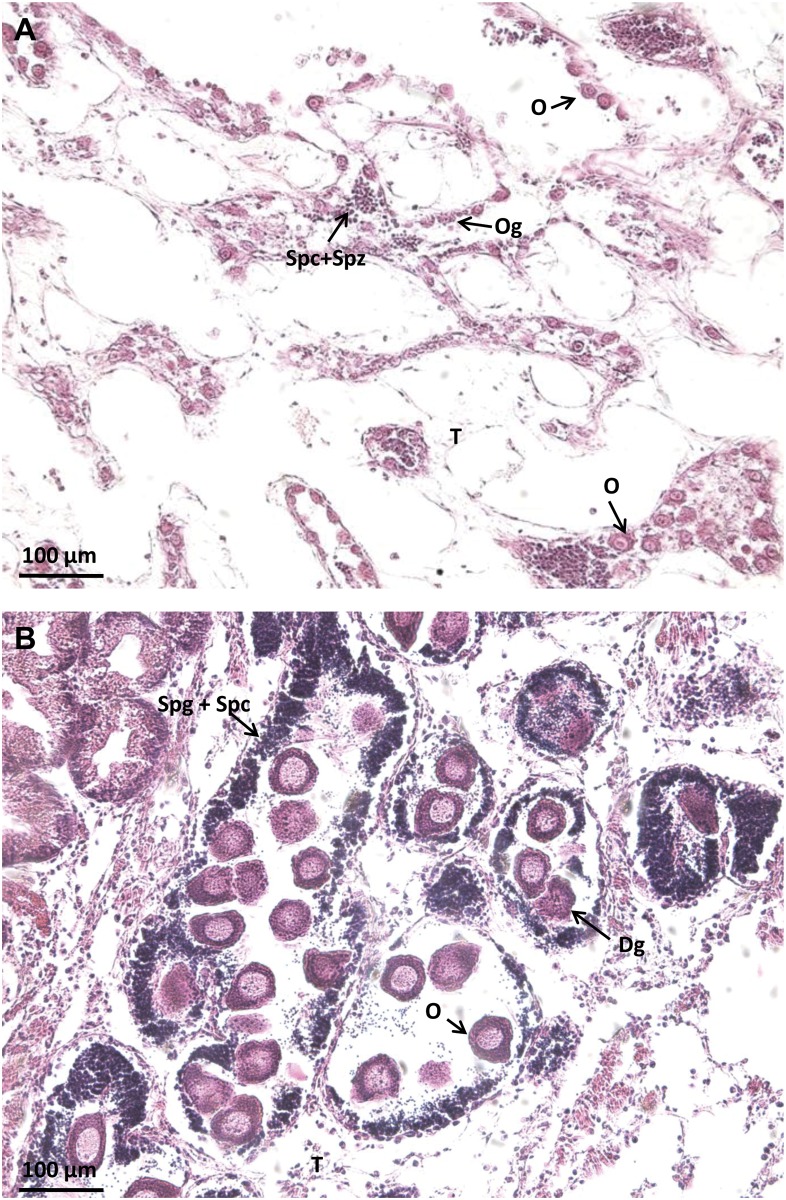
Histological features of gonads in sexual inversion in *P*. *margaritifera*. Two types of sexual inversion are distinguished: sexual inversion from male to female (InvF, Table 1) (A), and sexual inversion from female to male (InvM, Table 2) (B). Og: ovogonia; O: oocytes; Dg: degenerating oocytes; Spg: spermatogonia; T: connective tissue; Spc: spermatocytes; Spz: spermatozoids.

From the clustering analysis results coupled with histological analyses, we modified the previous classification of *P*. *margaritifera* gonads proposed in Teaniniuraitemoana *et al*. (2014) [29] to establish a new one, no longer based on a histological approach alone but on a dual histo-molecular analytical approach considering the gene expression pattern of gonads. This new classification consists of 16 gonadic categories and can be divided into two main groups: gonads in the male and the female pathway characterized by a male and a female gene expression pattern, respectively. These new gonadic categories are described in Tables 1 and 2. Fig. 2 is used to describe the two types of gonad in sex inversion, i.e. InvF and InvM.

**Table 1:** Histological description of the gonadic categories of the female sexual pathway of *P*. *margaritifera*.

**F** (female gametogenesis)	The gonad is in gametogenesis.
**FE** (early stage—developing)	Tubules are less bulky and contain mostly oogonia and oocytes in early development. Connective tissue is abundant.
**FI** (intermediate stage—developing)	Tubules are large. Oocytes in early development are still numerous and those at the end of vitellogenesis begin to accumulate. The connective tissue is less abundant.
**FM** (mature stage—ripe)	Tubules are turgid and contain only mature oocytes or those close to maturity.
**FR** (regression stage)	Tubules are retracted or retract. Oogonia are absent. Tubules contain some degenerating oocytes from the previous gametogenetic cycle.
**UndF** (undetermined—undifferentiated)	Tubules are retracted or absent. No germ cells.
**MRF** (male regression stage)	Tubules are retracted or retract. Spermatogonia are detached from the wall of tubules or completely absent. Residuals spermatozoids (with sometimes spermatocytes) from the previous gametogenetic cycle are found in the lumen.
**InvF** (sex inversion from male to female)	The female germline appears at the periphery of acini. Several oocytes in early development. Residual spermatozoids from the previous gametogenetic cycle are present in the center of tubules. (Fig. 2).

These gonadic categories have a female gene expression pattern at molecular level.

**Table 2:** Histological description of the gonadic categories of the male sexual pathway of *P*. *margaritifera*.

**M** (male gametogenesis)	The gonads is in gametogenesis.
**ME** (early stage—developing)	Tubules are less bulky and contain mostly spermatogonia and spermatocytes. Connective tissue is abundant.
**MI** (intermediate stage—developing)	Tubules are large. Spermatogonia and spermatocytes form a ring at the periphery of tubules. Spermatozoids occupy the central part. The connective tissue is less abundant.
**MM** (mature stage—ripe)	Tubules are turgid. Spermatozoids fill the entire lumen and spermatogonia and spermatocytes are very few.
**MR** (regression stage)	Tubules are retracted or retract. Spermatogonia are detached from the wall of tubules or completely absent. Residuals spermatozoids (with sometimes spermatocytes) from the previous gametogenetic cycle are found in the lumen.
**UndM** (undetermined—undifferentiated)	Tubules are retracted or absent. No germ cells.
**FRM** (female regression stage)	Tubules are retracted or retract. Oogonia are absent. Tubules contain some degenerating oocytes from the previous gametogenetic cycle.
**InvM** (sex inversion from female to male)	The male germline appears at the periphery of acini. Several spermatogonia and spermatocytes. Degenerating oocytes from the previous gametogenetic cycle are present. (Fig. 2).

These gonadic categories have a male gene expression pattern at molecular level.

### Selection of nine candidate genes of the sexual pathway in *P*. *margaritifera*

**Fig 3:**
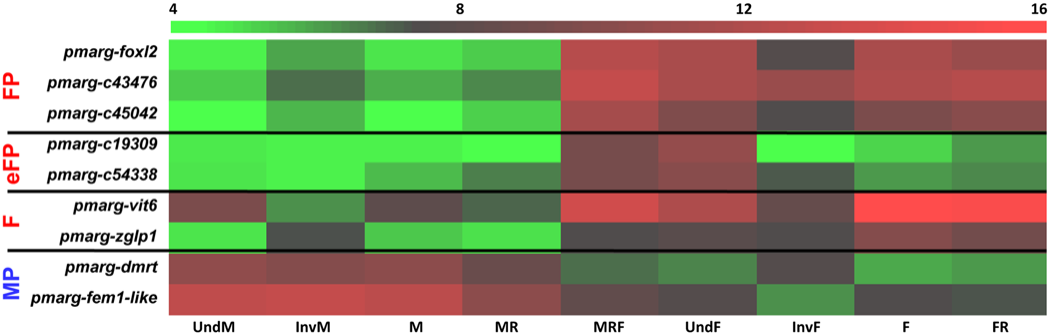
RNAseq expression of the nine selected genes of the sexual pathway of *P*. *margaritifera*. Heat map of the RNAseq expression in the new gonad categories of the nine selected genes. Color represents the normalized expression after variance-stabilizing transformation (DESeq). Expression levels are depicted with a color scale, in which shades of red represent higher expression and shades of green represent lower expression. FP: the overall female pathway, eFP: early female pathway, F: female in active gametogenesis, MP: the overall male pathway. M or F: male or female in gametogenesis; (M/F)R: male/female at regression stage; Und(M/F): Undifferentiated gonad at the cellular level beginning a male or a female determination/differentiation at the molecular level; Inv(M/F): oyster in sexual inversion female to male or male to female; MRF: male at regression stage at the cellular level beginning a female differentiation at the molecular level.

We performed a second differential expression analysis considering the gonadic categories from the new proposed histo-molecular classification. Thus, this second DE analysis was performed using the DESeq package between the M category and each of the categories of the female sexual pathway as follows: M *vs* F, M *vs* FR, M *vs* MRF, M *vs* IndF and M *vs* InvF. We then compared all the DE results in order to obtain the contigs commonly differentially expressed in all the comparisons described above. Nine genes were thus selected (Table 3). Three genes among these nine, *pmarg-foxl2* coding for the Forkhead box protein L2 (Contig_43072) and two unknown genes *pmarg-c43476* and *pmarg-c45042* (Contig_43476 and Contig_45042), were expressed at least tenfold more in MRF, UndF, InvF, F and FR oyster gonads than in male gonads. They were thus considered as markers of the entire female pathway (FP). Two other unknown genes, *pmarg-c19309* and *pmarg-c54338* (Contig_19309 and Contig_54338), were expressed at least tenfold more in MRF and UndF oyster gonads (i.e., oysters presented female gene expression pattern at the molecular level but no female germ cells are visible at the cellular level) than all other categories, and therefore represent markers of the “early” female pathway (eFP). Two genes coding for the GATA-type zinc finger protein 1 (*pmarg-zglp1*, Contig_25360) and vitellogenin-6 (*pmarg-vit6*, Contig_15150), showed expression at least tenfold higher in female oyster gonads in gametogenesis than in other categories, and thus were classed as markers of the female category (F). Finally, the two last genes, *pmarg-fem1-like* and *pmarg-dmrt* were expressed at least tenfold more in categories of the male pathway (UndM, InvM, M and MR), and consequently were considered as markers of the male pathway (MP). RNAseq expressions of these nine genes, classed into the new gonadic categories, are presented in Fig. 3.

**Table 3:** List of the candidate marker genes of the sexual pathway of *P*. *margaritifera*.

*P*. *margaritifera* contigs	Gene name	Protein name
**Female pathway (MRF + UndF + InvF + F + FR)**		
Contig_43072	*pmarg-foxl2*	Forkhead box L2
Contig_43476	*pmarg-c43476*	Unknown gene product
Contig_45042	*pmarg-c45042*	Unknown gene product
**Early female pathway (MRF + UndF)**		
Contig_19309	*pmarg-c19309*	Unknown gene product
Contig_54338	*pmarg-c54338*	Unknown gene product
**Female category (F = FE + FI + FM)**		
Contig_15150	*pmarg-vit6*	Vitellogenin-6
Contig_25360	*pmarg-zglp1*	GATA-type zinc finger protein 1
**Male pathway (UndM + InvM + M + MR)**		
Contig_639	*pmarg-dmrt*	Doublesex- and mab-3 related transcription factor
Contig_1317	*pmarg-fem1-like*	Fem1-like protein

M or F: male or female in gametogenesis; FE: female at early stage; FI: female at intermediate stage; FM: female at mature stage; (M/F)R: male/female at regression stage; Und(M/F): Undifferentiated gonad at the cellular level beginning a male or a female determination/differentiation at the molecular level; Inv(M/F): oyster in sexual inversion female to male or male to female; MRF: male at regression stage at the cellular level beginning a female determination/differentiation at the molecular level.

**Fig 4:**
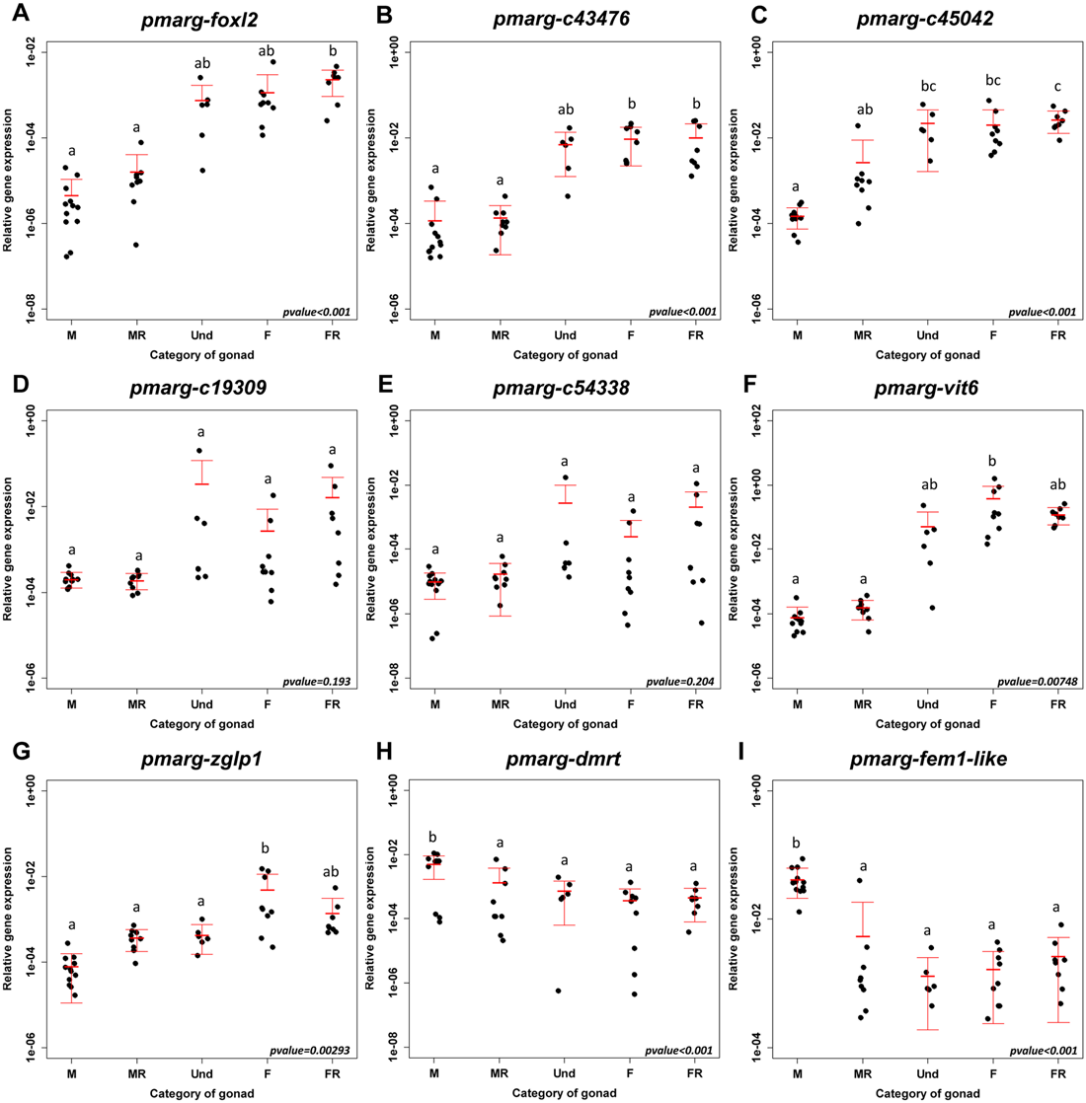
Relative expression of the nine selected marker genes of the female pathway of *P*. *margaritifera*. Relative expression profiles (real-time PCR) of *pmarg-foxl2* (A), *pmarg-c43476* (B), *pmarg-c45042* (C), *pmarg-c19309* (D), *pmarg-c54338* (E), *pmarg-vit-6* (F), *pmarg*-*zglp1* (G), *pmarg-dmrt* (H), and *pmarg-fem1-like* (I) in gonad categories (new set of samples). Different letters indicate statistically significant differences determined by ANOVA. M or F: male or female in gametogenesis; (M/F)R: male/female at stage of regression; Und: Undetermined sex.

To validate at the biological level these nine candidates as markers of the corresponding pathway, gene mRNA levels were then assessed using real-time PCR in a new set of oyster gonads (Fig. 4A-I). These samples were classified into five gonadic categories only: M, MR, Und, F and FR, because it was not possible to determine the stages MRF, UndF or UndM, and FRM using histology. Concerning FP candidate genes, results of relative expression showed a significantly higher expression in female gonads overall, especially for *pmarg-c43476* (F: 0.0099 ±0.0077; and FR: 0.0104 ±0.0107) (Fig. 4A, 4B and 4C). In the same way, relative expression of F and MP candidate genes showed levels equivalent to their expression found in the RNAseq dataset. Indeed, *pmarg-vit6* (0.39 ±0.53) and *pmarg-zglp1* (0.005 ±0.006) were significantly more expressed in F gonads (Fig. 4F and 4G), and *pmarg-dmrt* and *pmarg-fem1-like* were significantly more expressed in M gonads (0.005 ±0.004 and 0.04 ±0.02 respectively) (Fig. 4H and 4I). There was no significant difference between gonadic categories in the relative expression of early female pathway candidate genes (eFP), *pmarg-c19309* and *pmarg-c54338* (Fig. 4D and 4E).

### A 3-gene-pair expression ratio signature of the sexual pathway in *P*. *margaritifera*

**Fig 5:**
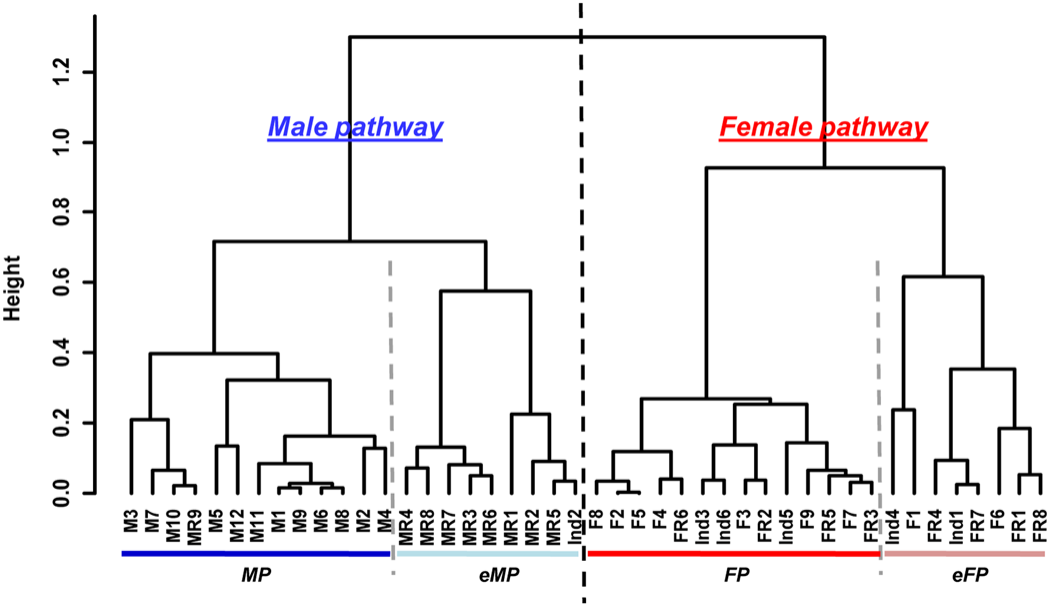
Hierarchical clustering using Spearman’s correlation on gonad samples. Samples are divided into two main clusters based on their gene expression ratio pattern (36 variables), discriminating male and female pathways. Each cluster is divided into two sub-clusters discriminating oysters in early male or early female pathway (eMP or eFP) and oysters in the course of male or female pathway (MP or FP). M or F: male or female in gametogenesis; (M/F)R: male/female at regression stage; Und: gonad sex undetermined.

To cluster samples according to their similarity of expression patterns by the nine candidate genes, we performed a hierarchical clustering using spearman correlation, based not on relative expressions (9 variables) but on their ratio (36 variables), as in *Ma et al*. (2004) [38]. Following this method, samples were grouped into two main clusters discriminating samples in the male and female pathways (Fig. 5). Moreover, each cluster was separated into two sub-clusters which divided oyster gonads on the early male or early female pathways (eMP or eFP), including undifferentiated and regressed gonads, from gonads in the course of active male or female gametogenesis (MP or FP).

**Fig 6:**
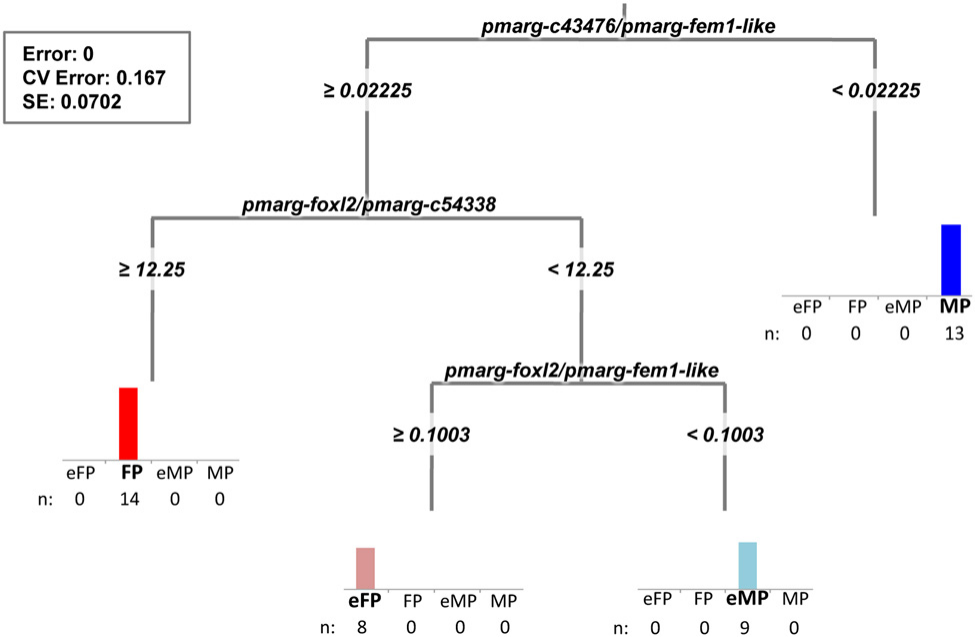
Multivariate regression tree of oyster sexual pathway categories. Candidate predictor/explanatory variables are the relative expression ratio of the nine potential marker genes (36 variables), and the response variables are the four sub-clusters of gonad samples (n = 44) determined by hierarchical clustering analysis (eMP or eFP: oyster on the early male or female pathway; MP or FP: oyster in the course of male or female pathway). This tree explained 100% of the total variance. The bar graphs at the end of each leaf represent the number of samples of the corresponding group. Statistics of the MRT are presenting at the left of the figure. Error: Residual error; CV Error: Cross-validation error; and SE: Standard error.

Next, to determine the major gene expression ratios explaining this clustering and thus having an effect on the sexual pathway, we performed a multivariate regression tree. A four-leaf MRT (where “leaf” refers to each terminal node), shown in Fig. 6, best described sexual pathways, explaining 100% of the total variance (Error = 0, Cross-Validation error = 0.167, Standard error = 0.0702). From the MRT model obtained, it appeared that, among the 36 input variables, the most important factors/predictors were three relative gene expression ratios implicating four candidate marker genes: *pmarg-c43476*, *pmarg-foxl2*, *pmarg-c54338* and *pmarg-fem1-like*. The first regression tree split, which explains 43% of the variance, is based on the relative gene expression ratio of *pmarg-c43476* to *pmarg-fem1-like*. If the expression ratio of these two genes is lower than 0.02 then, following the regression tree to the right, this means that the oyster sample is on the male pathway. When this ratio is higher than 0.02, following the MRT to the left, a second split then occurs based on the relative gene expression ratio of *pmarg-foxl2* to *pmarg-c54338*, which explains 30% of the variance. Samples showing *pmar-foxl2*/*pmarg-c54338* expression ratios greater or equal to 12.2 are on the female pathway, whereas samples showing a ratio lower than this critical value are on the early sexual pathway. Finally, the third and the last split of the regression tree (27% of the variance), is based on the *pmarg-foxl2*/*pmarg-fem1-like* expression ratio, and differentiates samples on the early female pathway (eFP) from those on the early male pathway (eMP) by a critical value of 0.1. Thus, following this MRT model of the sexual pathway of *P*. *margaritifera*, we can associate oysters with the particular pathways by analyzing the expression ratios of these four genes (i.e., *pmarg-c43476*/*pmarg-fem1-like*, *pmarg-foxl2*/*pmarg-c54338* and *pmarg-foxl2*/*pmarg-fem1-like*).

To test the predictive ability of this MRT model of the sexual pathway of *P*. *margaritifera* on other datasets, we predicted the sexual pathways on which were engaged the RNAseq samples. To do this, we used the RNAseq normalized expression (*estimatesizefactor*, DESeq) of the samples. The regression tree prediction matched with the previous hierarchical clustering (Fig. 1). Samples of the male cluster were predicted to be on the male pathway and samples in the female cluster were predicted to be on the female pathway as shown in Table 2. The undetermined oyster gonads (“Und1” and “Und2), oyster gonads in sexual inversion (“Inv1” and “Inv2”) and the male regressed gonad sample “MR2”, were classified as in the earlier analysis (Table 4, Fig. 1).

**Table 4:** Results of the predictive MRT model on RNAseq sample (data from [29]).

Sample		Cellular (histology)		Molecular	
*Bank ID*	*CAH ID*	*Sex*	*Stage*	*CAH cluster*	*Decision tree group*
17	ME1	Male	Early	Male	eMP
19	ME2	Male	Early	Male	MP
21	ME3	Male	Early	Male	MP
24	ME4	Male	Early	Male	MP
4	MI1	Male	Intermediate	Male	MP
18	MI2	Male	Intermediate	Male	MP
22	MI3	Male	Intermediate	Male	MP
30	MI4	Male	Intermediate	Male	MP
78	MM1	Male	Mature	Male	MP
123	MM2	Male	Mature	Male	MP
134	MM3	Male	Mature	Male	MP
135	MM4	Male	Mature	Male	MP
42	MR1	Male	Regression	Male	eMP
**63**	**MR2**	**Male**	**Regression**	**Female**	**eFP**
69	MR3	Male	Regression	Male	eMP
130	MR4	Male	Regression	Male	MP
8	FE1	Female	Early	Female	FP
31	FE2	Female	Early	Female	FP
43	FE3	Female	Early	Female	FP
133	FE4	Female	Early	Female	FP
27	FI1	Female	Intermediate	Female	FP
56	FI2	Female	Intermediate	Female	FP
83	FI3	Female	Intermediate	Female	FP
138	FM1	Female	Mature	Female	FP
143	FM2	Female	Mature	Female	FP
144	FM3	Female	Mature	Female	FP
148	FM4	Female	Mature	Female	FP
64	FR1	Female	Regression	Female	FP
79	FR2	Female	Regression	Female	eFP
132	FR3	Female	Regression	Female	FP
147	FR4	Female	Regression	Female	FP
**1**	**Inv1**	**Inversion**	**-**	**Female**	**FP**
**150**	**Inv2**	**Inversion**	**-**	**Male**	**MP**
**57**	**Und1**	**Undetermined**	**-**	**Female**	**eFP**
**71**	**Und2**	**Undetermined**	**-**	**Male**	**MP**

## Discussion

As marine bivalves are organisms of major economic interest, genomic research has recently been conducted to provide greater insight into the molecular mechanisms governing various complex physiological traits [39–44]. Among those, several genomic studies have been employed successfully to better understand cellular and molecular mechanisms involved in reproductive behavior, including gametogenesis, sex determinism and sex differentiation [25–27,45,46]. Recently, in a previous study, a gonad transcriptome analysis of *P*. *margaritifera* was performed using Illumina-based RNAseq, which allowed to elucidate some molecular mechanisms involved in sex determination, sex differentiation and gametogenesis in this protandrous hermaphrodite species [29].

Here, we re-analyzed the quantification data of the *de novo* gonad transcriptome of *P*. *margaritifera* to identify marker genes of the sexual pathway and therefore acquire further insights into the complex and unusual reproduction of the black-lip pearl oyster.

### A dual approach to modeling *P*. *margaritifera* reproduction

Although hierarchical clustering, based on the expression pattern of the 1,937 contigs determined as differentially expressed using a stringent statistical analysis, did not discriminate gametogenic stages, a clear grouping of the different sexes was observed. This marked and specific gonadic gene expression had already been shown for *P*. *margaritifera* [29] as well as for other marine bivalves with different reproductive functioning, including the Pacific oyster *Crassostrea gigas* (an irregular and alternative protandrous hermaphrodite) [25], Pacific lion-paw scallop *Nodipecten subnodosus* (a functional hermaphrodite) [26], and European clam *Ruditapes decussatus* (a gonochoric species) [27].

Interestingly, it appeared that individuals undergoing sexual inversion were distributed in both male and female clusters. Although these individuals undergoing sex inversion and presented gametes of both sexes, the main gene expression pattern resulted from the sex that an oyster would become. This molecular aspect is in accordance with the cellular aspects found by observation of histological sections of gonads in the course of sexual inversion. Indeed, the new germ line, consisting of several oocytes or spermatocytes, appears at the periphery of the acini, whereas the former sex phenotype is represented only by a few residual spermatozoids or degenerated oocytes [9]. Moreover, in the hierarchical clustering, one of the two undifferentiated individuals was grouped on the same branch as the females, and the other on the same branch as the males. Misclassification has already been observed in two other marine bivalve species, *C*. *gigas* and *R*. *decussatus*, where individuals at undetermined sex stages were found to express male- or female-specific genes, suggesting that sex differentiation had already taken place, even if it was not visible at cellular level using histology [25,27]. Here, the most unexpected result was the finding of an individual histologically determined as a regressed male grouped with female oysters. This result, leads us to hypothesize that although this oyster could be determined as male due to the presence of residual spermatozoids, it had already initiated female sex differentiation according to its gene expression pattern. Following this result, regression stage appears to constitute the sex-switching time window in *P*. *margaritifera*, as already proposed in *C*. *gigas*. For this latter irregular alternative hermaphrodite species, the sex-determining time window may occur in the period around the end of a reproductive cycle and the beginning of the next [20].

**Fig 7:**
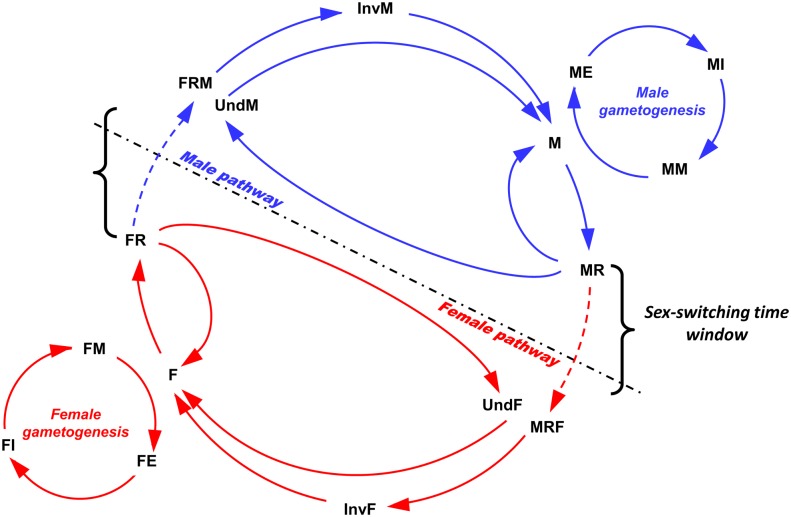
The histo-molecular model of the reproduction of *P*. *margaritifera*. M or F: male or female in gametogenesis; (M/F)E: male/female at early stage; (M/F)I: male/female at intermediate stage; (M/F)M: male/female at mature stage; (M/F)R: male/female at regression stage; Und(M/F): gonad undifferentiated at the cellular level, beginning a male or a female differentiation at the molecular level; Inv(M/F): oyster in sexual inversion female to male or male to female; MRF: male in regression stage at the cellular level beginning a female differentiation at the molecular level; and FRM: female in regression stage at cellular level beginning a male differentiation at the molecular level.

All these results reveal the importance of considering both histological and molecular, approaches in the description and the understanding of the atypical reproduction of *P*. *margaritifera*. Indeed, these previous results allow us to propose a kinetic of the gonadic sex and stages found in the male and the female sexual pathway of this species, modeled in Fig. 7.

This is the first histo-molecular reproduction model proposed for a marine bivalve species, taking into account molecular signatures coupled with cellular descriptions. Thus, at the end of a gametogenetic cycle, female or male gonads (M or F) would pass through a regression phase (MR or FR) before starting a new cycle. This gonad regression occur after a spawning event as other marine bivalve species [47–49] but might also occur when food are not abundant. Indeed, *P*. *margaritifera* gametogenesis is known to be modulated by food level and it was showed recently that the under-feeding decreases or stops the germinal activity preventing any resumption of gametogenesis [11]. From this regression stage, gonads might also completely regressed and become undetermined (UndM or UndF) before starting a new gametogenetic cycle. Here, oysters are certainly in sexual rest. Gonads are undifferentiated at cellular level but shows early molecular signs of differentiation allowing us to know in which sexual pathway the oysters are. Moreover, as mentioned above, regression stage appears the sex-switching time window of *P*. *margaritifera*. Indeed, the transition from the male to the female pathway seems to occur from the gonad regression of male oysters and the inverse for the transition from the female to the male pathway. Indeed, male or female oysters pass through a regression stage (MRF/FRM) before reaching an inversion stage (InvF/InvM) to become females or males at gametogenesis. The factors involve in this sex change are not well known in *P*. *margaritifera*. In *C*. *gigas*, interaction between environmental, as temperature, and genetic factors could be involved in sex determination and thus in sex inversion [14,15,19]. In our bivalve species, Thielley (1993) [9] showed that changes from female to male could occur when environmental conditions (temperature or food) were stressful, but no study has yet shown environmental or genetic factors inducing changes from male to female. This proposed model of the reproduction of *P*. *margaritifera* will help to elucidating these mechanisms.

### The nine identified marker genes reveal a 3-gene-pair expression ratio model of the sexual pathway in *P*. *margaritifera*

Mechanisms of sex determination, which are critical for development and reproduction, are among the least well-known processes in biology, since they vary a great deal among species, in both primary signal and downstream genetic pathways [50,51]. Sex determination is a crucial question that still needs to be elucidated in marine bivalves, especially pearl oyster, because of important implications for aquaculture. Providing a repertoire of molecular markers of the sexual pathway is essential for further studies on sex determination for this protandrous hermaphrodite bivalve.

Here, we selected nine genes as potential markers of the sexual pathway based on their differential RNAseq expression, considering the new gonadic categories of the histo-molecular classification. There were four unknown genes whose role cannot yet be interpreted, and five genes functionally assigned by orthology: *pmarg-foxl2*, *pmarg-vit6*, *pmarg-zglp1*, *pmarg-dmrt* and *pmarg-fem1-like*. These latter genes have already been shown to play a role in sex determination or sex differentiation in other organisms. *pmarg-foxl2*, coding for the Forkhead box protein L2, has previously characterized and discussed in the pearl oyster [29]. In mammals, *foxl2* is expressed in follicular cells and is the earliest known marker of ovarian differentiation [52,53]. The two genes *pmarg-vit6* and *pmarg-zglp1* coding for vitellogenin-6 and the GATA-type zinc finger protein 1, respectively, were identified here as putative markers of the female pathway. Indeed, vitellogenin, a female specific glycoprotein constituting the yolk protein, was identified as being necessary for building oocytes [25,54]. The GATA-type zinc finger protein 1, also named GATA-like protein-1 (GLP-1), was identified to be required for normal fertility in female mice, as its deficiency leads to the absence of oocytes at birth [55]. Finally, the two genes *pmarg-dmrt* and *pmarg-fem1-like*, orthologs of family genes known to be involved in sex differentiation and sex determination respectively in model organisms and previously discussed in Teaniniuraitemoana *et al*. (2014) [29], are underlined here for the male pathway.

To go beyond the limits of histological analysis mentioned above, we considered the 44 new gonadic samples based on their relative marker gene expression ratios. Hierarchical clustering revealed that oyster gonad samples can be clustered into four groups differentiating, on a first level, oysters on the main male and female pathways, and on a second level, oysters in the early stages of the male or female pathways. Herein, we successfully identified three gonad-expressed gene ratios correlated with the oyster sexual pathway. Among the 36 gene ratios, three were enough to explain 100% of the variance and thus can be considered as major sexual determinants. This 3-gene-pair ratio sexual pathway signature is *pmarg-c43476*/*pmarg-fem1-like*, *pmarg-foxl2*/*pmarg-c54338* and *pmarg-foxl2*/*pmarg-fem1-like*. It implicates two genes defined as potential markers of the female pathway, *pmarg-c43476* and *pmarg-foxl2*; one unknown gene, *pmarg-c54338*, detected as a marker of the early female pathway; and *pmarg-fem1-like*, a marker of the male pathway. Interestingly, the level (0.1) of the gene expression ratio between *pmarg-foxl2* and *pmarg-fem1-like* appears crucial in the sex-switching and sex differentiation of *P*. *margaritifera* distinguishing the early female and early male pathways. The *P*. *margaritifera fem1-like* gene was characterized as a derived member of the *Fem1* family, which includes the *fem-1* gene required for normal masculinization of somatic and germline tissue in the worm *Caenorhabditis elegans* [56]. The high expression of *pmarg-fem1-like* might inhibit the expression of the *pmarg-foxl2* and thus cause the male differentiation of the oyster. Such a pattern can be observed in *C*. *elegans*: *fem* genes inhibit *tra-1*, which would otherwise produce the protein TRA-1A whose sequence-specific DNA-binding would otherwise bring about female sex differentiation by regulating the transcription of target genes [57,58]. These previous results strongly suggest also that the *P*. *margaritifera foxl2* gene might not only be involved in early ovary differentiation but also in ovary maintenance. Indeed, its large over-expression throughout the time course of the female pathway could suggest that *pmarg-foxl2* might restrain the genetic program for testis differentiation by active repressing of *pmarg-fem1-like* gene. This maintenance mechanism involving *foxl2* was already demonstrated in mammals by Uhlenhaut *et al*. (2009) [59]. The authors showed that the mammalian ovarian phenotype has to be maintained throughout adulthood, mainly by a down regulation of the Sertoli cell-promoting gene *Sox9* expression which Foxl2, coupled with estrogen receptors, is the critical factor responsible [59]. Although our results are insufficient to conclude that *pmarg-fem1-like* and *pmarg-foxl2* are major sex-determining genes in *P*. *margaritifera*, they would certainly play a key role in the molecular cascade of sex differentiation at a downstream level. Furthermore, unknown genes detected with relevant expression would be of great interest for further studies, as shown in other aquatic organisms [60].

## Conclusion

The most significant outcomes of our study are the two models we constructed allowing us to improve our understanding of the specific reproduction of the marine bivalve *P*. *margaritifera*. The first is a model based on two approaches of the reproduction in this species defining the gonadic stage occurring in the sexual pathway by considering two levels of analysis: histological and molecular. In particular it reveals that the sex-inversion time window of *P*. *margaritifera* occurs during a regression phase of the gonad.

The second is a 3-gene-pair expression ratio based model, which makes it possible to predict the sexual pathway in this hermaphrodite species and can thus be used as a tool for future studies on sex inversion, sex differentiation and sex determination in *P*. *margaritifera*. Such work will aim to control reproduction for selection programs, thus supporting the sustainable development of pearl farming in French Polynesia. This latter model also revealed the importance of the expression of *pmarg-foxl2* and *pmarg-fem1-like* for sex inversion and sex differentiation and the need to elucidate their role in *P*. *margaritifera* based on further functional research, such as the use of RNA interference now available in marine bivalves [61].

## Supporting Information

S1 Table Primer sequences. (XLSX)
